# Supplementary material for: Tuning of task-relevant stiffness in multiple directions
Source: Sci Rep. 2025 Aug 15;15:29916. doi: 10.1038/s41598-025-14989-8 (PMC12356977; doi:10.1038/s41598-025-14989-8)
Supplement: Supplementary file 1 — Supplementary Material 1 [file 41598_2025_14989_MOESM1_ESM.docx]

SUPPLEMENTARY MATERIAL FOR

Tuning of Task-Relevant Stiffness
in Multiple Directions

Chenguang Zhang^1^, Federico Tessari^3^, James Hermus^3^, Himanshu Akolkar^2^, Neville Hogan^3,4^, Andrew B. Schwartz^1,2*^

^1^Dept. of Bioengineering, ^2^Dept. of Neurobiology, Univ. of Pittsburgh, Pittsburgh, PA; ^3^Dept. of Mechanical Eng., ^4^Dept. of Brain and Cognitive Sci., MIT, Cambridge, MA; ^5^Robot Learning & Interaction Group, Idiap Research Institute, Martigny, Switzerland

*Corresponding Author: Dr. Andrew Schwartz ([abs21@pitt.edu](mailto:abs21@pitt.edu))

1. *General subject information*

| Subject # | Gender | Height(cm) | Weight(kg) |
| --- | --- | --- | --- |
| 1 | M | 170.2 | 63.2 |
| 2 | M | 175.0 | 65.7 |
| 3 | M | 182.9 | 85.8 |
| 4 | M | 160.0 | 50.2 |
| 5 | M | 183.0 | 82.0 |
| 6 | M | 172.0 | 77.8 |
| 7 | M | 180.0 | 90.0 |
| 8 | M | 183.0 | 78.5 |
| 9 | M | 182.0 | 81.3 |
| 10 | F | 163.0 | 60.0 |
| 11 | M | 180.0 | 75.0 |
| 12 | M | 182.0 | 113.0 |
| 13 | M | 188.0 | 101.5 |
| 14 | F | 163.0 | 55.2 |
| 15 | M | 180.3 | 65.0 |
| 16 | F | 162.6 | 62.1 |
| 17 | F | 160.0 | 58.0 |
| 18 | F | 157.5 | 58.5 |
| 19 | F | 168.0 | 57.3 |
| 20 | F | 162 | 46.4 |

1. *Success rate across subjects*

Success rate was defined as the number of successful trials (each subject needed to perform nine successful trials to complete each block) divided by the total number of trials in which the subject managed to complete the force hold stage of the task. Varying success rates were observed across subjects (Supplementary Figure 1) with a mean across directions of 64.10% $\pm19\%$. One-way repeated measure ANOVA detected a statistically significant effect of direction on success rate $(F_{3,76}=4.12, p= 0.0092)$. Post-hoc pairwise comparisons showed a statistically significant difference between the +X and -Y direction ($p = 0.00$8), while no significant difference was present between other directions.


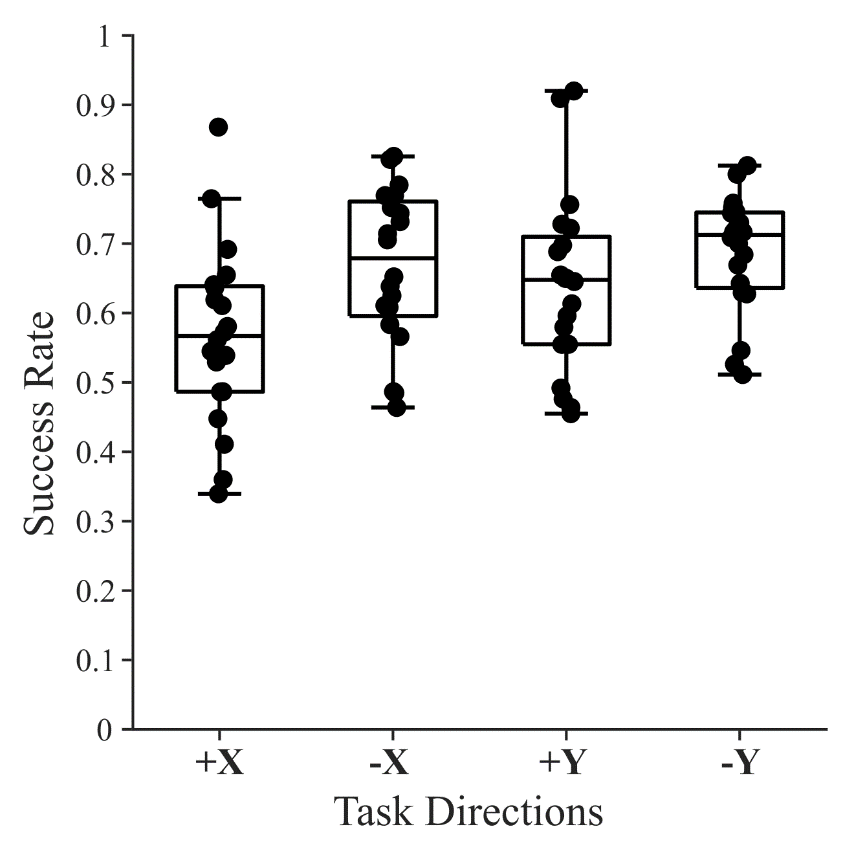


Supplementary Figure 1 – The success rate in different task directions. Dots show the task success rate of each subject. The box plot shows the median and the 25^th^ and 75^th^ percentile performance across subjects in each direction.

1. *2^nd^ Order Model Fitting Performance*

Supplementary Table 1 reports the average fitting performance (FIT%) across trials and subjects in the 9 different conditions and 4 different directions.


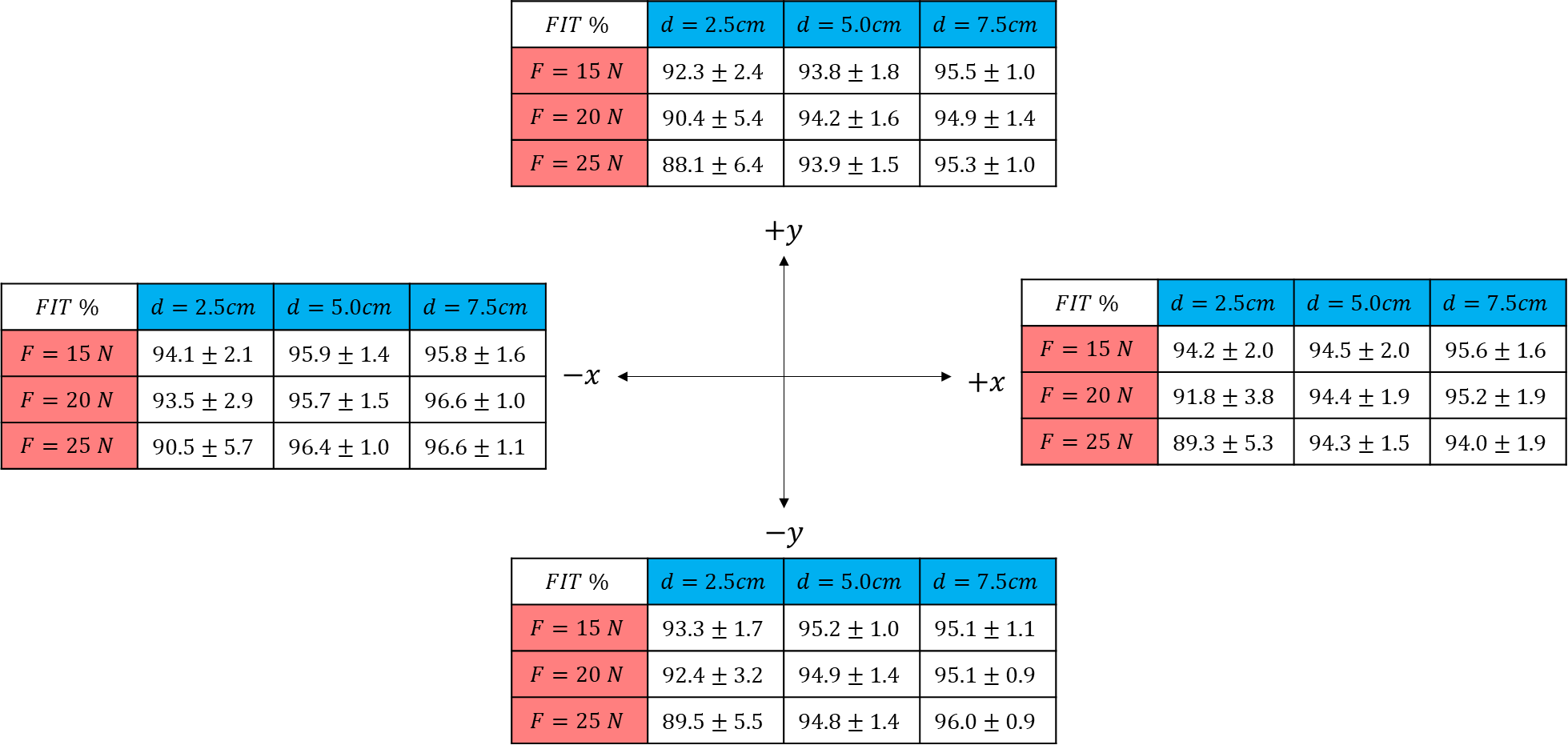


Supplementary Table 1 – The four different tables present the fitting performance across trials and subjects in the 9 force-displacement tested conditions for each movement direction. The reported numbers represent the mean and standard deviation of the fitting performance FIT%.

Supplementary Figure 2 shows the sensitivity of the FIT% to changes of $\pm30\%$of the identified impedance parameters ($k_{est},\beta_{est},m_{est}$). In order to simplify the interpretation of sensitivity analysis, the estimated impedance values – as well as the FIT% – were normalized for each subject, direction, and condition.

Supplementary Figure 2 – Sensitivity analysis of the normalized fitting performance (FIT%) to $\pm30\%$ variations of the estimated impedance parameters ($k_{est},\beta_{est},m_{est}$) obtained from the 2^nd^ order model system identification.

Supplementary Figure 3 reports the fitting performance differences between successful and failed trials as well as the difference in the stiffness estimates across all subjects and directions. The results confirm that a 2^nd^ order model was able to competently describe the arm end-effector behavior in all cases.


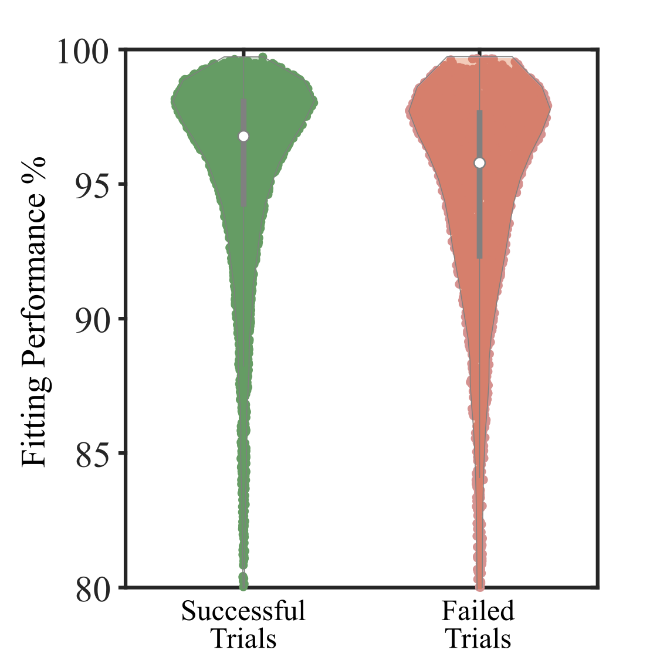

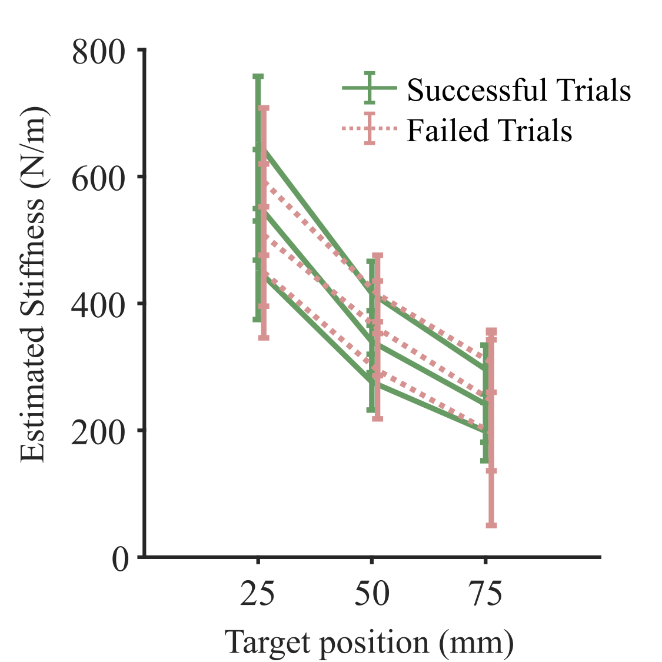


Supplementary Figure 3 – Fitting performance for the 2^nd^ order model was found to be high for data from all trials irrespective of whether the subjects succeeded or failed. The estimated stiffness from the failed trials followed the same trend as the successful trials. This shows that the model could still fit the data. Trials were removed from the analysis not because the model did not fit but because subjects failed to comply with the task requirements.

Supplementary Figure 4 reports the estimated impedance parameters (mass, spring and damping) for a representative subject.


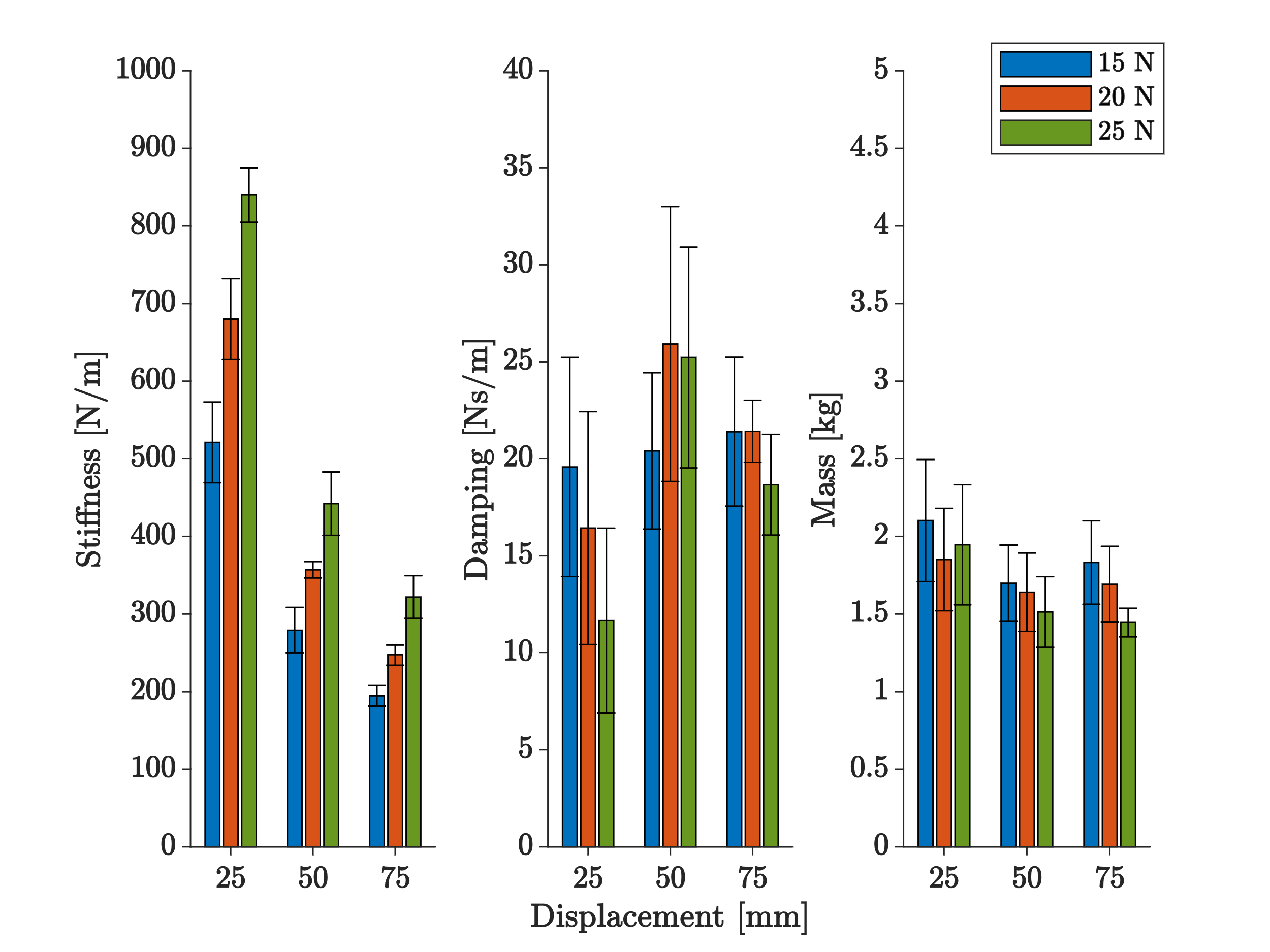


Supplementary Figure 4 – Estimated impedance parameters for a representative subject. For each panel, the x-axis shows the 3 different target displacements, while the 3 different colors represent the different target forces. Error bars indicate the standard deviation about the mean. The stiffness estimates (left panel) increased as force increased and decreased as the target displacement increased. In the middle panel, the damping coefficient appeared not to show any particular trend with respect to the different conditions. In the right panel, the mass was largely constant across different conditions.

Supplementary Figure 5 shows the estimated stiffness for all trials and all subjects for directions -X and -Y. The results, similar to Figure 8, show how subjects generated a task stiffness lower than the nominal task stiffness (solid blue bar) and close to the minimum allowed value required by the task.


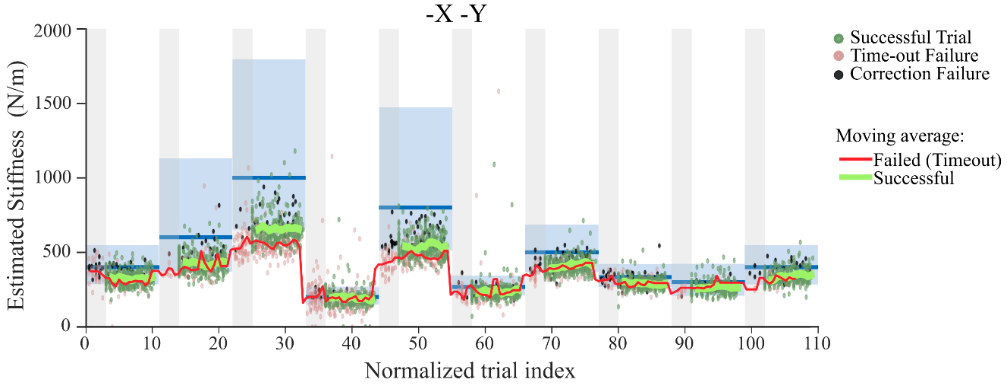


Supplementary Figure 5 – Estimated stiffness for all trials across sessions for directions -X and -Y (corresponding to +X, +Y directions shown in Figure 8). The figure follows the same format as Figure 8. The conditions were presented in the same order for these directions. White sections show the trials between the first and last successful trials in a condition. Gray sections show the failed trials between the last successful trial of one condition and the first successful trial of the next condition. Dots represent the estimated stiffness for each trial where green represents a successful trial, red represents failure due to time-out and black dots show failed trials due to corrections during movement. The blue bars show the range of nominal stiffness, $k_{n}=\frac{F_{tg}}{d_{tg}}$ and the blue rectangle shows the range of stiffness based on the allowed force and displacement tolerances. The red line shows the average estimated stiffness for failed (time-out) trials whereas the green solid lines show the average estimated stiffness for successful trials.

1. *Maximum Voluntary force*

We collected subjects’ maximum voluntary force in different movement directions, calculated as the average force for 10s during which the subject was instructed to exert maximum isometric force. Maximum voluntary force varied from 46.1N to 211N, with forces in the left and back directions generally higher than in the forward and right directions (Supplementary Figure 6A). Each subjects’ maximum voluntary force was greater than the highest force condition (25N) in the task. Maximum voluntary force was weakly but positively correlated (Supplementary Figure 6B) with body mass (R=0.5187).


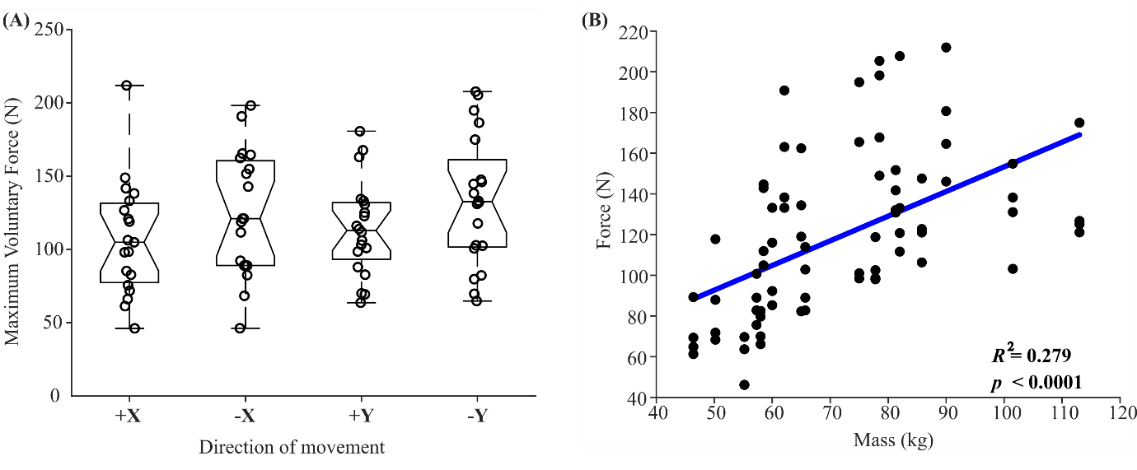


Supplementary Figure 6 – Maximum voluntary force. **A.** Maximum voluntary force across directions. Left and back direction forces were slightly higher than right and forward. This might be because subjects had stronger pectoralis and biceps, which primarily exerted left and back forces, compared to posterior deltoid and triceps, which primarily generated right and forward forces. **B.** Maximum voluntary force varied with subject mass. Subjects with greater mass tended to exert higher maximum voluntary force. The plot also reports the linear $R^{2}$ regression coefficient and a p-value, which indicated a slope significantly different from zero.

1. *Stiffness, Damping and Mass Estimates*

A two-way repeated measures ANOVA was performed to analyze the effect of force and displacement on stiffness, with trials as repeated measures and direction as a random factor. The analysis revealed that there was a statistically significant interaction between the effects of force and displacement $(F_{4,675}=226.77, p<0.001)$. This interaction is evident in the top four plots of Figure 4, by the slight change in slope between the three target positions. Simple main-effects analysis showed that displacement had a statistically significant effect on stiffness in all pair-wise comparisons ($p<0.05$). The same analysis applied to force showed that it also had a statistically significant effect on stiffness in all pair-wise comparisons ($p<0.05$). These two effects are substantial and visibly evident in Figure 4 by the clear separation between the force and direction conditions. Supplementary Table 2 reports the complete post-hoc pairwise comparisons.

Nine two-way repeated-measures ANOVAs were performed to analyze the effect of direction on stiffness in the different force-displacement conditions. Trial was the repeated measure. The analysis revealed that there was a statistically significant effect ($p<0.05$) of direction in all force-displacement conditions. Supplementary Table 3 reports the details of ANOVA results and all post-hoc pairwise comparisons. While significant, these differences were not substantial; the differences in estimated stiffness with respect to direction were contained within the range of expected target stiffness (light blue area in Figure 7) and accounted – on average – for only $8\%\pm4\%$ of the minimum expected stiffness.

The top plots in Supplementary Figure 7 report the damping factor estimates across subjects in four different directions, while the bottom plots report the damping estimates in the nine different conditions with respect to movement direction.

Four two-way repeated measure ANOVAs were performed to analyze the effect of force and displacement on damping in the different directions. Trial was the repeated measure. The analysis revealed that there was a statistically significant interaction between the effects of force and displacement in all directions: +X$(F_{4,162}=37.57, p<0.001)$, -X $(F_{4,162}=24.34, p<0.001)$, +Y $(F_{4,162}=50.10, p=0.015)$, -Y $(F_{4,162}=19.47, p<0.001)$. Simple main effects analysis showed that displacement had a statistically significant ($p<0.05$) effect on damping in 81% of the pair-wise comparisons. Simple main effects analysis showed that force had a statistically significant ($p<0.05$) effect on damping in 69% of the pair-wise comparisons. The Supplementary Material, Section I, reports the complete post-hoc pairwise comparisons in Supplementary Tables 4,5,6, and 7.

Nine two-way repeated-measure ANOVAs were performed to analyze the effect of direction on damping in the different force-displacement conditions. Trial was the repeated measure. The analysis revealed that there was a statistically significant effect ($p<0.05$) of direction in all force-displacement conditions. Post-hoc pairwise comparisons showed that direction had a statistically significant ($p<0.05$) effect on damping estimates in 85% of the pair-wise comparisons. The Supplementary Material, Section I, reports the detailed ANOVA results and complete post-hoc pairwise comparisons in Supplementary Table 8.

The results show a high variability in the damping factor estimates, which appear noisy and without particular trend with respect to any of the analyzed parameters: direction of motion, target force, and target displacement. The counterintuitive significance can be explained by computing a linear fit of the damping factor estimates with respect to force and displacement. The 95% confidence intervals of the damping factor slope (with respect to both force and displacement) included zero in all but one case (23/24 slopes). This confirmed a high variability and insubstantial change of damping factor with respect to task conditions.


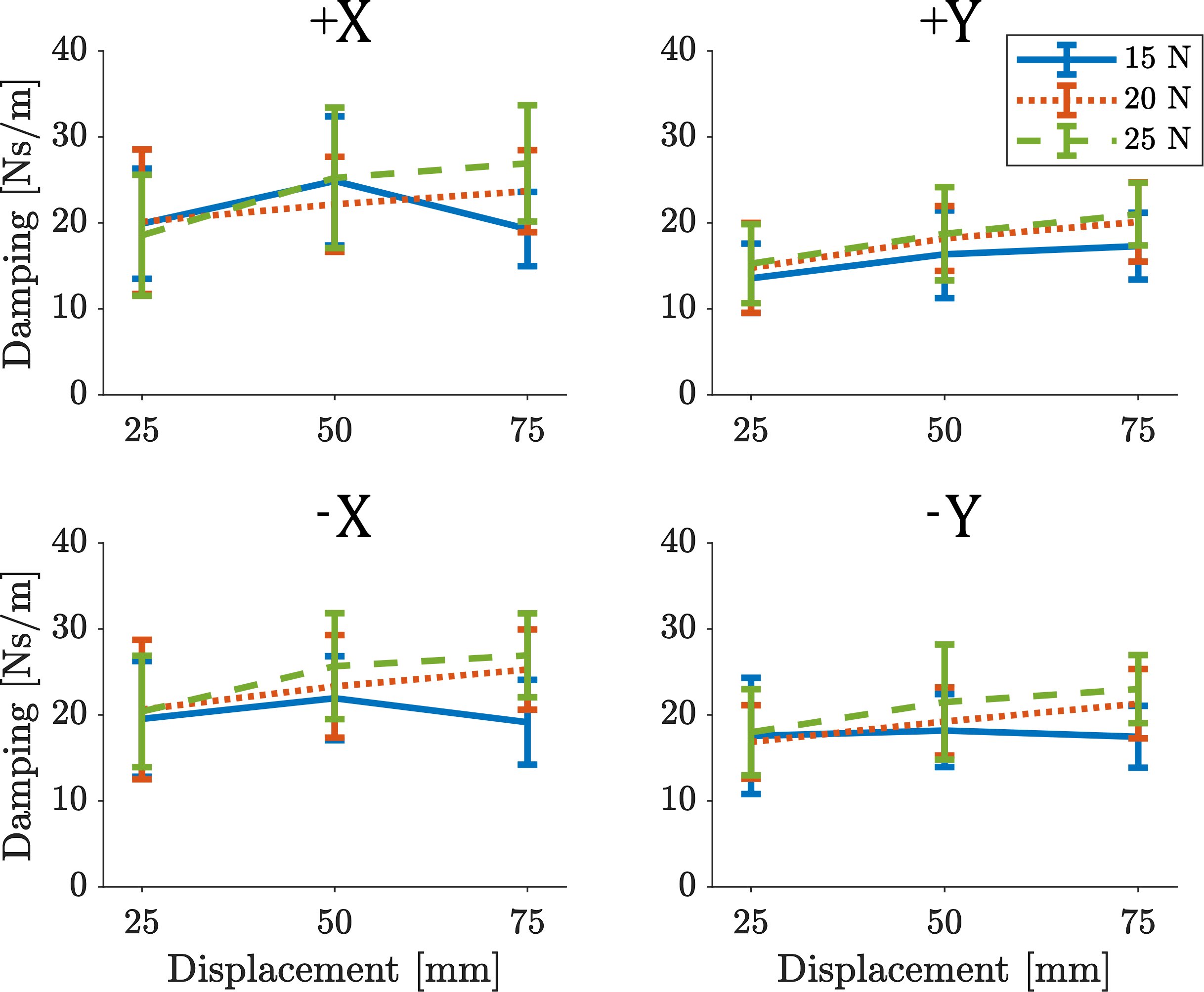


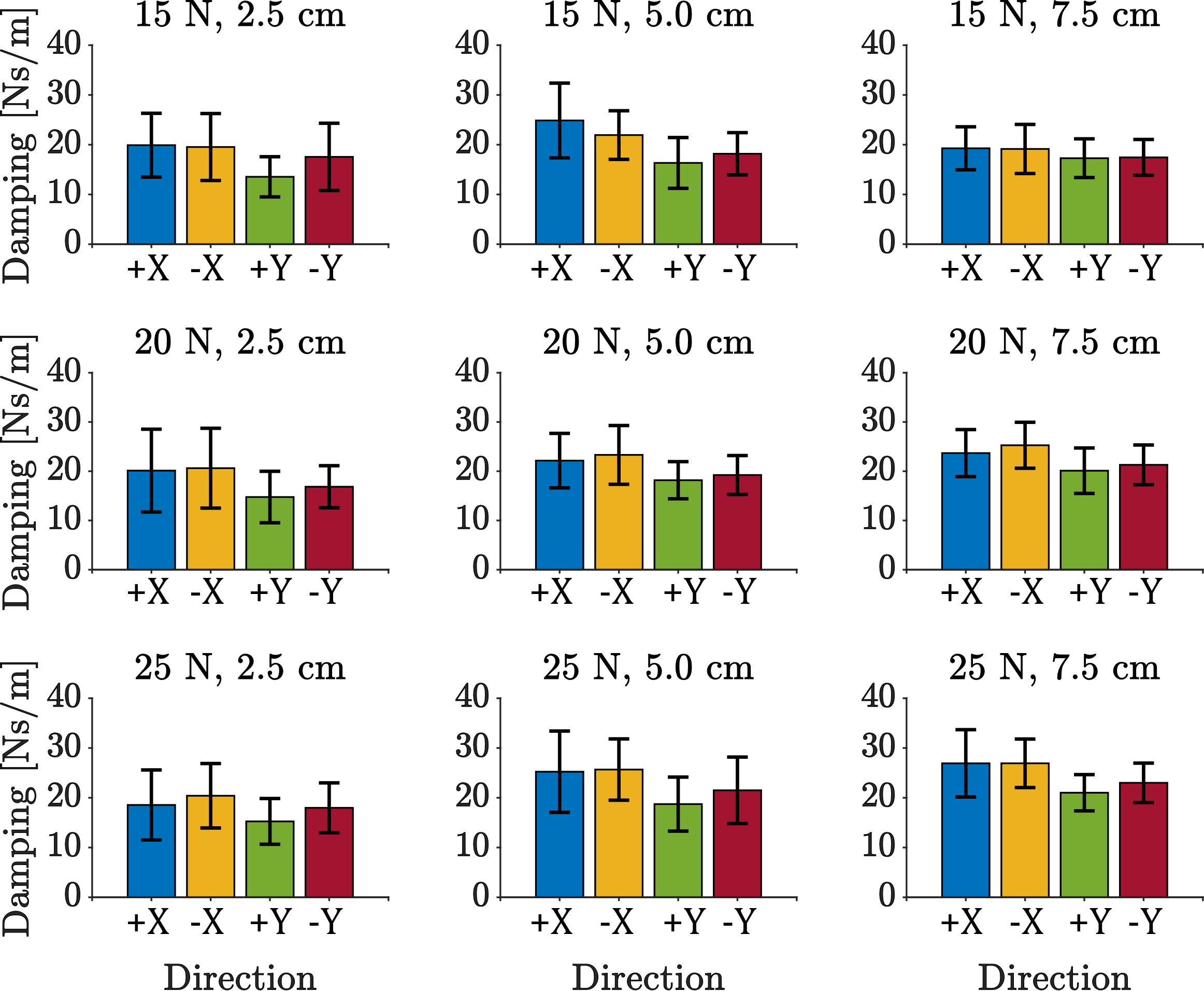


Supplementary Figure 7 – Arm damping variation across subjects. The top group of plots (4 plots) shows the damping coefficient change with respect to force and displacement in the four directions: +X (top left), +Y (top right), -X (bottom left), -Y (bottom right). For each panel, the 9 different force-displacement conditions are presented: the x-axis shows the different target displacements, 3 colored lines represent the different target forces. The bottom group of plots (9 plots) shows the damping change in the different force-displacement conditions with respect to the 4 directions of motion. The error bars in both plots show one standard deviation. (Complementary to Figure 7 in the main text with the same format).

To average the mass parameters across subjects, each estimated mass was normalized with respect to the subject’s body weight. The estimated end-effector mass is a percentage of overall body weight. The normalized mass estimates in the four directions are shown in Supplementary Figure 8.

Four two-way repeated measure ANOVAs were performed to analyze the effect of force and displacement on mass in different directions. Trial was the repeated measure. The analysis revealed that there was a statistically significant interaction between the effects of force and displacement in all directions: +X$(F_{4,162}=18.72, p<0.001)$, -X $(F_{4,162}=22.12, p<0.001)$, +Y $(F_{4,162}=14.24, p<0.001)$, -Y $(F_{4,162}=16.77, p<0.001)$. Simple main effects analysis showed that displacement had a statistically significant ($p<0.05$) effect on mass in 81% of the pair-wise comparisons. Similarly, force had a statistically significant ($p<0.05$) effect on mass in 47% of the pair-wise comparisons. The Supplementary Material, Section I, reports the complete post-hoc pairwise comparisons in Supplementary Tables 9,10,11, and 12.

Nine two-way repeated measure ANOVAs were performed to analyze the effect of direction on mass in the different force-displacement conditions. Trial was the repeated measure. The analysis revealed that there was a statistically significant effect ($p<0.05$) of direction in all force-displacement conditions. Post-hoc pairwise comparisons showed that direction showed a statistically significant $(p<0.05)$ difference in mass estimates in 96% of the pair-wise comparisons. The Supplementary Material, Section I, reports the ANOVA detailed results and complete post-hoc pairwise comparisons in Supplementary Table 13.

Similar to the damping factor analysis, a linear fit of the mass estimates with respect to force and displacement showed a rate of change (slope) 95% Confidence Interval that included zero slope in all but three cases (21/24 slopes). This indicated that the mass estimates were statistically invariant with respect to target displacement and target force. A significant difference with respect to motion direction was evident. Specifically, the mass in the lateral direction ($\pm X$) was significantly higher than in the ($\pm$Y) frontal direction. This difference is attributable to the orientation of the hand mass ellipsoid, which is due to the arm inertia (refer to the Supplementary Material, Section G).


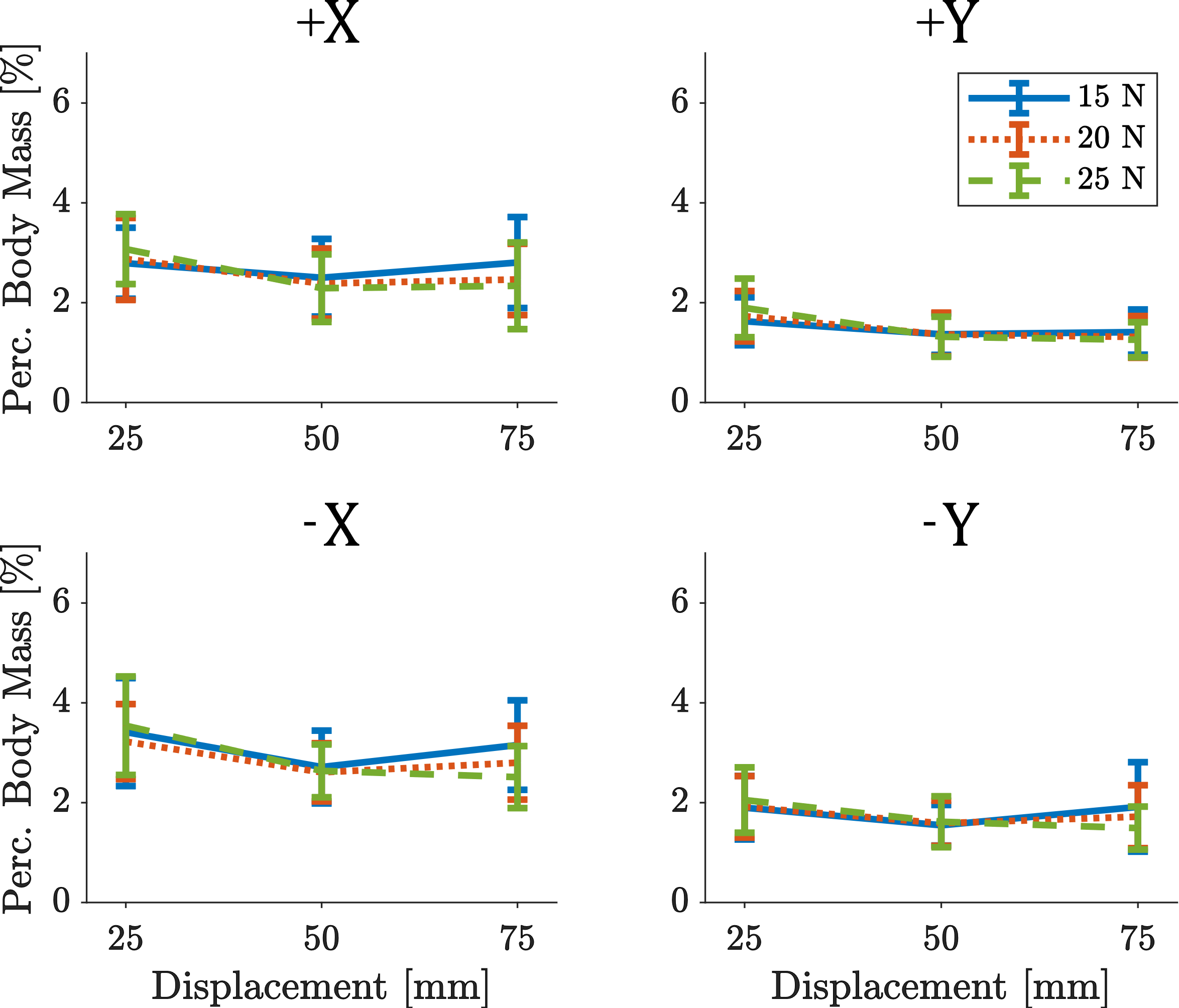


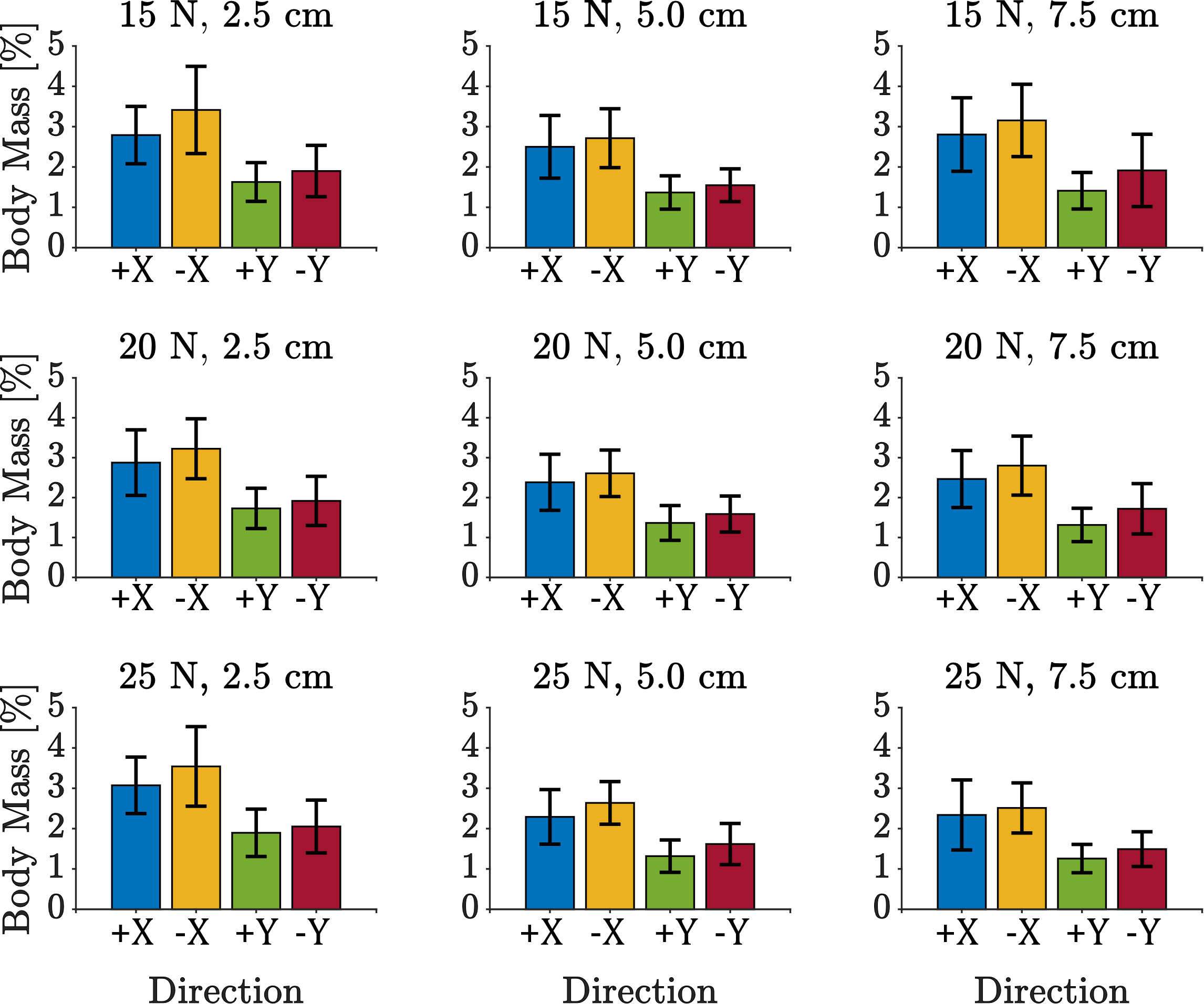


Supplementary Figure 8 – Arm end-effector mass variation across subjects. The top group of plots (4 plots) shows the mass coefficient (normalized by subject body weight) change with respect to force and displacement in the four directions: +X (top left), +Y (top right), -X (bottom left), -Y (bottom right). For each panel the 9 different force-displacement conditions are presented: the x-axis shows the different target displacements, 3 colored lines represent the different target forces. The bottom group of plots (9 plots) shows the mass change in the different force-displacement conditions with respect to the 4 directions of motion. The error bars in both plots show one standard deviation. (Complementary to Figure 7 in the main text with the same format).

1. *Two Degree of Freedom Arm Model*

A two DoF arm model was developed to analyze the effect of configuration changes on the end-effector mass and stiffness ellipses. The model, presented in Supplementary Figure 9, consisted of two rigid links representing the upper-arm and the forearm connected by means of simple rotational joints representing respectively the shoulder and elbow joints. The model considers the inertial properties of the human arm extracted from Winter (*54*). It did not include the gravitational load since the experiments were carried out in a plane perpendicular to the gravity vector and the arm was supported by a sling.

The equations of motions of such a system can be obtained by applying the Lagrange Equations, thus obtaining what follows:

$$M\left( \boldsymbol{\theta} \right)\ddot{\boldsymbol{\theta}}\boldsymbol{+}C\left( \boldsymbol{\theta},\dot{\boldsymbol{\theta}} \right)\mathcal{= F}$$

Where:

$$M\left( \boldsymbol{\theta} \right)= \left[ \begin{matrix} m_{1}L_{1G}^{2}+m_{2}\left( {L_{1}^{2}+2L}_{1}L_{2G}\cos\left( \theta_{2} \right)+L_{2G}^{2} \right)+I_{1} & m_{2}\left( L_{1}L_{2G}\cos\left( \theta_{2} \right)+L_{2G}^{2} \right) \\ m_{2}\left( L_{1}L_{2G}\cos\left( \theta_{2} \right)+L_{2G}^{2} \right) & m_{2}L_{2G}^{2}+I_{2} \end{matrix} \right]$$

$$C\left( \boldsymbol{\theta,}\dot{\boldsymbol{\theta}} \right)= \left[ \begin{matrix} -m_{2}L_{1}L_{2G}\left( 2\dot{\theta}_{1}\dot{\theta}_{2}+\dot{\theta}_{2}^{2} \right)\sin\left( \theta_{2} \right) \\ m_{2}L_{1}L_{2G}\dot{\theta}_{1}^{2}\sin\left( \theta_{2} \right) \end{matrix} \right]$$

$$\mathcal{F=}\left[ \begin{matrix} \frac{\partial W}{\partial q_{1}} \\ \frac{\partial W}{\partial q_{2}} \end{matrix} \right]$$

The geometrical and inertial quantities are summarized in the following list:

- Geometrical Quantities
  - $L_{1} \to$ Shoulder-Elbow distance (experimentally measured)
  - $L_{2} \to$ Elbow-Hand distance (experimentally measured)
  - $L_{1G}=0.436L_{1} \to$Upper-arm Center of Mass (CoM) position (*54*)
  - $L_{2G}=0.682L_{2}$ $\to$Forearm Center of Mass (CoM) position (*54*)
- Inertial Quantities (*54*)
  - $m_{1}=0.028M \to$ Upper-arm Mass with respect to Body Mass $M$
  - $m_{2}=0.022M$ $\to$ Forearm Mass with respect to Body Mass $M$
  - $I_{1}=m_{1}\rho_{1G}^{2} \to$ Upper-arm Moment of Inertia with respect to CoM
  - $I_{2}=m_{2}\rho_{2G}^{2} \to$ Forearm Moment of Inertia with respect to CoM
  - $\rho_{1G}=0.322L_{1} \to$ Upper-arm Radius of Gyration
  - $\rho_{2G}=0.468L_{2} \to$ Forearm Radius of Gyration


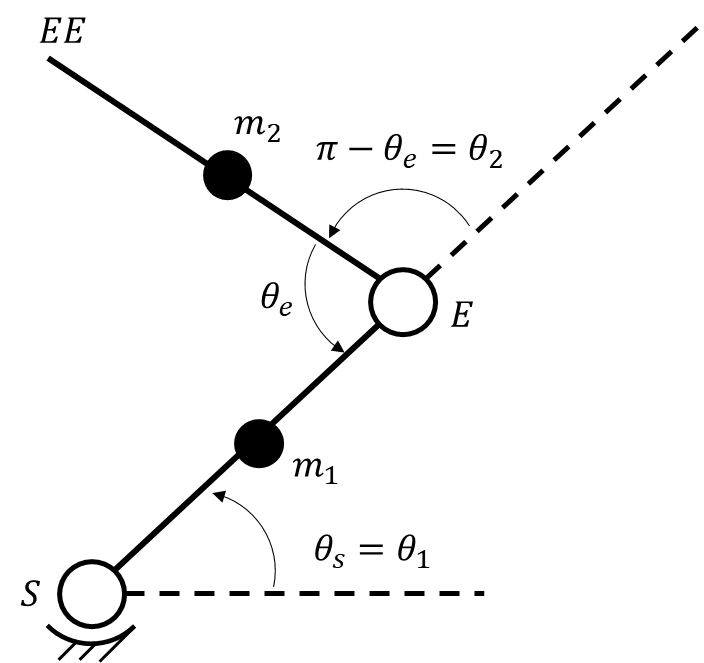


Supplementary Figure 9 – Schematic of the 2 DoF model of the arm. 'S' represents the shoulder joint, ‘E’ the elbow joint, ‘EE’ the position of the end-effector, ‘$\theta_{s}=\theta_{1}$’ the shoulder angle, and ‘$\theta_{e}$’ the elbow angle. $m_{1}$ is the mass of the upper arm, while $m_{2}$ is the mass of the forearm.

The mass matrix $M(\boldsymbol{\theta})$ represents the inertial properties of the model and how it varies over configuration space i.e., the joint space. The same matrix can be expressed in terms of workspace coordinates, which in our case will be the end-effector planar coordinates (x,y), thus leading to the so-called end-effector mass matrix. From a mathematical standpoint, this is obtained as:

$$\Lambda\left( \boldsymbol{\theta} \right)=J^{-T}(\boldsymbol{\theta})M(\boldsymbol{\theta})J^{-1}(\boldsymbol{\theta})$$

Where $\Lambda\left( \boldsymbol{\theta} \right)$ is the end-effector or spatial mass matrix and $J(\boldsymbol{\theta})$ is the Jacobian matrix computed as follows:

$$J\left( \boldsymbol{\theta} \right)\boldsymbol{=}\left[ \begin{matrix} \boldsymbol{-}L_{1}\sin\left( \theta_{1} \right)-L_{2}\sin(\theta_{1}+\theta_{2}) & -L_{2}\sin(\theta_{1}+\theta_{2}) \\ \boldsymbol{-}L_{1}\cos\left( \theta_{1} \right)-L_{2}\cos(\theta_{1}+\theta_{2}) & -L_{2}\cos(\theta_{1}+\theta_{2}) \end{matrix} \right]$$

In a similar way, the joint stiffness $R$ can be transformed to the end-effector stiffness matrix exploiting the Jacobian matrix. However, in this specific case, the joint stiffness matrix will also need to take into account the so-called kinematic stiffness $\Gamma=\frac{\delta J^{T}\left( \theta\right)}{\delta\theta}F$, which accounts for the apparent stiffness generated by external forces $F$(*10*).

Consequently, end-effector stiffness can be computed as:

$$K(\boldsymbol{\theta})={J\left( \boldsymbol{\theta} \right)}^{-T}\cdot\left( R-\Gamma\right)\cdot{J(\boldsymbol{\theta})}^{-1}$$

1. *(1) Stiffness tuning does not arise from bio-mechanical configuration*

As mentioned in the Introduction, there are two contributions to endpoint force and stiffness: one passive, due to the configuration of the arm, the other active, due to changes in muscle excitation. In this subsection, we modeled the variation of passive endpoint stiffness due to joint configuration and found that it was much smaller than the stiffness measured during the task. The active muscle component completely dominated our results.

To estimate the variation of endpoint stiffness due to biomechanical configuration, the model combined a constant joint impedance i.e., no active control, with the joint position collected by the motion-tracking system (Optotrak 3020, Northern Digital, Canada) as input, and returned as output the resulting endpoint stiffness. In simpler terms, we positioned the 2-DoF arm model in a posture equivalent to the one used in the experiments. Then, we fixed a constant stiffness at each joint – thus simulating a passive muscle condition – and finally simulated displacing the end-effector a unit distance $(x,y)$ in each direction. We used the end-effector displacement in combination with the constant joint stiffness to estimate the joint torques and from them calculate the endpoint forces $(F_{x},F_{y}).$ The distribution of the resulting forces on the plane forms an ellipse, from which we found the stiffnesses $\left( K_{x},K_{y} \right)$.


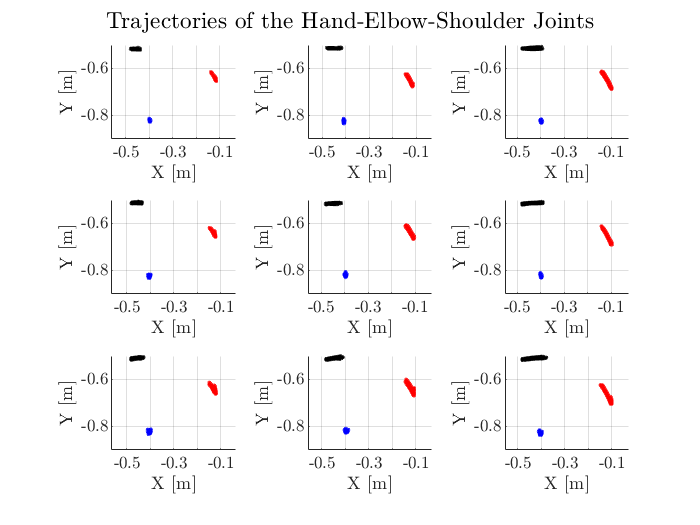


Supplementary Figure 10 – Trajectories of the hand (black dots), elbow (red dots), and shoulder (blue dots) markers in the horizontal (x,y) plane for a representative subject. The 9 panels represent the nine different force-displacement conditions performed during the ballistic release experiment. The target force increases from the first to the last row, while the target displacement increases from the first to the last column.

We computed the predicted endpoint stiffness as a series of ellipses, depending on the different joint locations, and plotted the start (force hold) and final (position hold) locations with black and red separately. The stiffness ellipses were generated by imposing a unit planar circle displacement $(x,y)$ around an equilibrium configuration in the 2-DoF model, and by calculating the resulting forces $(F_{x}, F_{y})$. From those data we computed stiffness in both the movement direction and orthogonal to it, $K_{x}$, $K_{y}$.

Supplementary Figure 11 shows the resulting endpoint stiffness ellipses (force hold in black, and position hold in red) in each of the 9 force-displacement conditions. Two key aspects emerge. First, moving from the force hold (black ellipse) to the position hold (red ellipse) introduced a small stiffness variation, ranging from 5% in the smallest force, shortest displacement case, to 20% in the biggest force, largest displacement case. These differences confirm that the change in arm configuration – from force to position hold – within a given condition did not have a strong influence on the stiffness behavior. This is likely due to the fact that the experimentally applied forces were sufficiently small (<30 N), and the displacements sufficiently limited (<10 cm).

Second, considering the stiffness ellipses across the 9 conditions, we observed that the variations in endpoint stiffness due to the change in force and displacement were particularly limited. In the force-hold phases (black ellipses), the endpoint stiffness maximum difference across conditions is 6% in $K_{x}$, and 6.9% in $K_{y}$. Similarly, in the position hold phases (red ellipses), the endpoint stiffness maximum difference across conditions is 8.7% in $K_{x}$, and 7.5% in $K_{y}$.

These results confirm our hypothesis that biomechanical configuration i.e., arm position, had a negligible influence on the observed changes in stiffness during the ballistic release experiment. In fact, the ballistic release experiment showed measured endpoint stiffness differences – across conditions – of more than 110%.

In other words, to render such variations in endpoint stiffness, subjects have to actively tune their joints stiffness, rather than simply relying on the passive effects of biomechanical configuration of the arm.

Supplementary Figure 11 – The endpoint stiffness prediction in different task conditions obtained using the two degree of freedom arm model. The 9 panels represent the 9 nine different force-displacement conditions performed during the ballistic release experiment. The target force increases from the first to the last row, while the target displacement increases from the first to the last column. For each panel two ellipses are presented: the black ellipse represents the end-effector stiffness ellipse during force hold, while the red ellipse shows the end-effector stiffness ellipse during position hold. The small line segments in each panel are included to show the difference in position of the arm model. On top of each panel the values of the resulting end-effector stiffness in both the X (lateral) and Y (frontal) directions, respectively K_x_ and K_y_, are reported both during the force hold phase (t_0_) and in the final position hold phase (t_f_).

1. *(2) The endpoint mass is sufficiently constant and in agreement with the 2^nd^ order model.*

The endpoint mass was estimated in the same way. Each body segment mass and center of mass was estimated from participants’ height and weight. For mass coefficients, the model took each subject’s body-mass index (BMI) and joint positions as input and returned the end-effector mass as output (each body segment mass was assumed according to Winter (*54*)). The endpoint mass was calculated as described in Section F. The ellipses were generated by imposing a planar unit circle of acceleration $(\ddot{x},\ddot{y})$ and measuring the resulting forces $(F_{x}, F_{y})$. The ellipses from different conditions only changed with different movement positions. The fact that the mass prediction from the kinematic model overlapped the mass coefficient estimated from the 2^nd^-order model validates the estimates from our 2^nd^-order model.

Supplementary Figure 12 – The endpoint mass predictions in different task conditions were obtained using the two degree of freedom arm model. The 9 panels represent the 9 nine different force-displacement conditions performed during the ballistic release experiment. The target force increases from the first to the last row, while the target displacement increases from the first to the last column. For each panel two ellipses are presented: the black ellipse represents the end-effector mass ellipse during force hold, while the red ellipse shows the end-effector mass ellipse during position hold. The small line segments in each panel are added to show the difference in position of the arm model. On top of each panel the values of the resulting end-effector mass in both the X (lateral) and Y (frontal) directions, respectively M_x_ and M_y_, are reported both during the force hold phase (t_0_) and in the final position hold phase (t_f_).

1. *EMG data analysis*

Supplementary Figure 13A shows the average normalized EMG across all subjects from the three antagonist muscle pairs during the force hold phase of the task for the highest stiffness condition in the four task directions. The wrist muscles were active in all directions whereas the Biceps-Triceps and the deltoids were active only in certain directions. Moreover, the CAI was found to be strongly positively correlated with stiffness when the muscles were active (Supplementary Figure 13B).


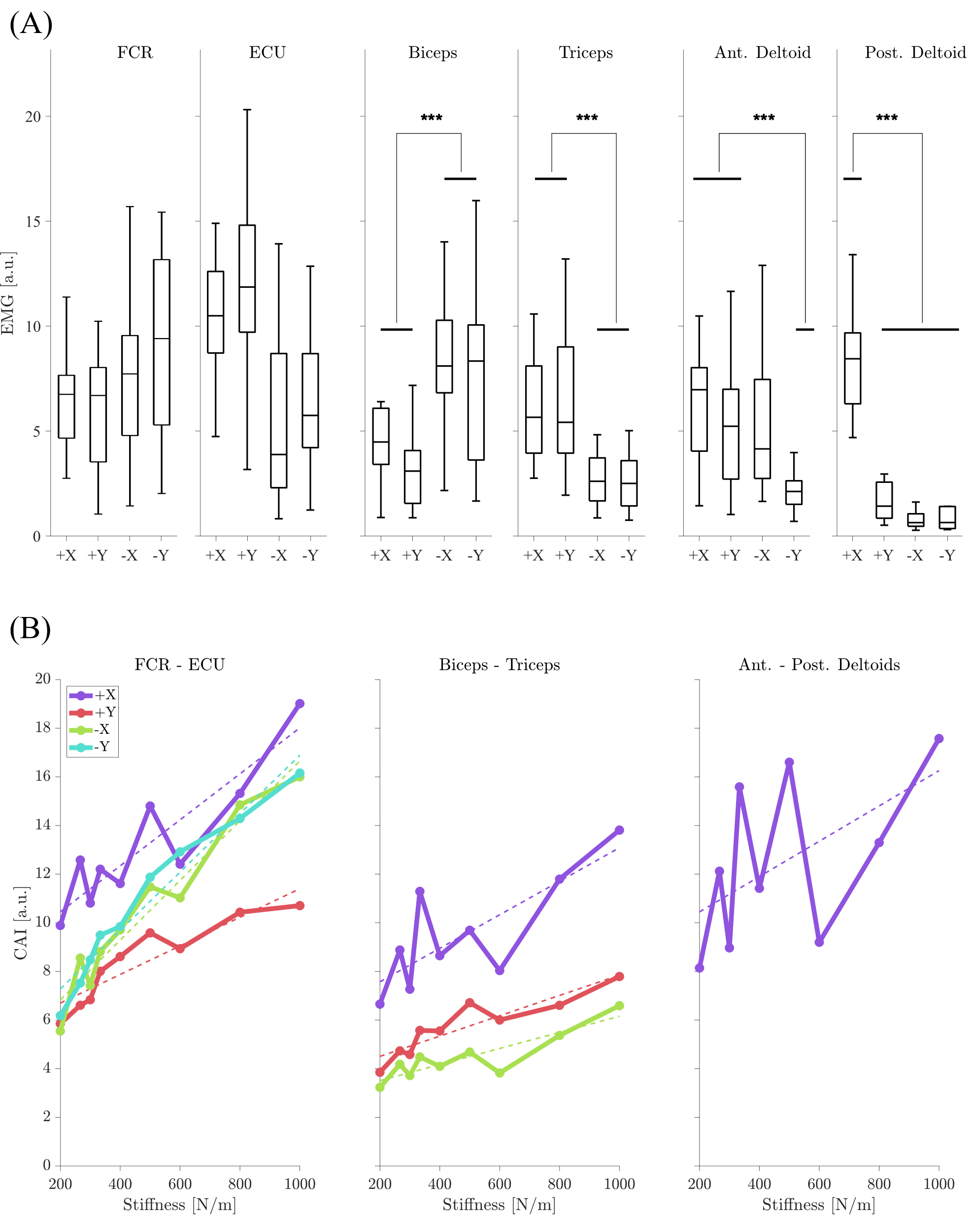


Supplementary Figure 13 **-** EMG dependency on movement direction. **A.** Average EMG across all subjects from the force-hold phase for all muscles for the four task directions. The FCR and ECU were active in all directions. Asterisks indicate statistical significance of the difference in activity across directions (***: p < 0.001). The biceps and triceps were active in opposite directions. The anterior deltoid was active in three directions, while the posterior deltoid was active only in one direction. **B.** CAI of different muscle pairs for the directions in which the muscles pairs were active. The CAI was strongly and positively correlated with stiffness in the active directions.

Recorded EMG from the terminal position hold phase of the task was used to compute the co-activation index (CAI), which was then regressed against the three task parameters (K, F, and X) across all subjects and directions. Similar to the pre-movement phase, the co-activation of wrist muscles was found to be strongly correlated with stiffness but only weakly with force or displacement.


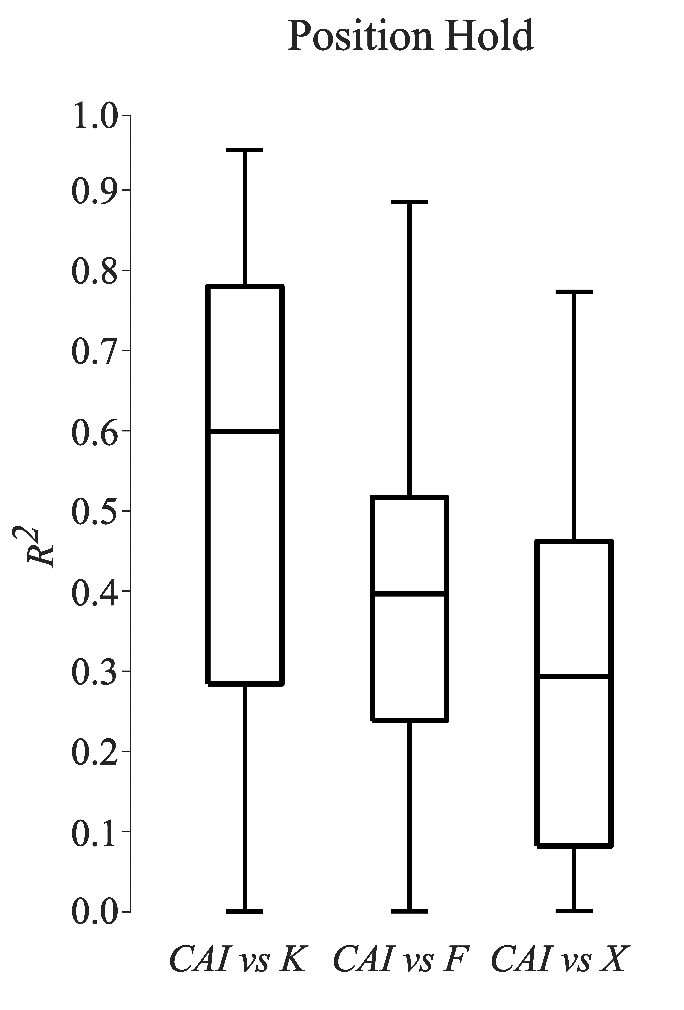


Supplementary Figure 14 – Co-activation index from average EMG activity recorded during the terminal position hold phase of the task was regressed against the various task parameters (similar to those shown in Figures 9D and Figure 10D). We found that the goodness of fit increased with the force-hold phase levels, especially for the stiffness parameter, suggesting that subjects maintained their co-activation of wrist muscles even when they reached the target location.

1. *Post-hoc t-tests Complete Results*

The post-hoc pairwise comparisons were performed when significant interaction was observed in any of the performed repeated measure ANOVAs. The following is a complete report of the pairwise comparisons divided per dependent variable.

*Stiffness*

*Effect of Target Force and Target Displacement*

Supplementary Table 2 - Pairwise comparisons of stiffness estimates with respect to target force and target displacement. The table reports the Bonferroni adjusted p-values for multiple comparisons. The significance was set to p=0.05. The left part of the table reports the comparisons between target displacements at the different force levels. The right part of the table reports the comparison between target forces at different displacement levels. The asterisk * denotes a significant difference.

|  | d=2.5cm/5.0cm | d=5.0/7.5cm | d=2.5cm/7.5cm |  | F=15N/20N | | F=20N/25N | | F=15N/25N | |
| --- | --- | --- | --- | --- | --- | --- | --- | --- | --- | --- |
| F=15N | *p*<0.001* | *p*<0.001* | *p*<0.001* | **d=2.5cm** | *p*<0.001* | *p*<0.001* | | *p*<0.001* | |  |
| F=20N | *p*<0.001* | *p*<0.001* | *p*<0.001* | **d=5.0cm** | *p*<0.001* | *p*<0.001* | | *p*<0.001* | |  |
| F=25N | *p*<0.001* | *p*<0.001* | *p*<0.001* | **d=7.5cm** | *p*<0.001* | *p*<0.001* | | *p*<0.001* | |  |

*Effect of Direction*

Supplementary Table 3 - This table reports the repeated measure ANOVA results (second column) and the post-hoc pairwise comparisons of stiffness estimates with respect to direction. The pair-wise comparisons were Bonferroni adjusted for multiple comparisons. The significance was set to p=0.05. The asterisk * denotes a significant difference.

|  | ANOVA | (+X, -X) | (+X, +Y) | (+X, -Y) | (-X, +Y) | (-X, -Y) | (+Y, -Y) |
| --- | --- | --- | --- | --- | --- | --- | --- |
| (15N,2.5cm) | $F=27.01$ $p<0.001$* | *p*<0.001* | *p*=1.000 | *p*<0.001* | *p*<0.001* | *p*=0.013* | *p*<0.001* |
| (15N,5.0cm) | $F=46.78$ $p<0.001$* | *p*<0.001* | *p*=0.035* | *p*<0.001* | *p*<0.001* | *p*<0.001* | *p*<0.001* |
| (15N,7.5cm) | $F=56.15$ $p<0.001$* | *p*<0.001* | *p*=1.000 | *p*<0.001* | *p*<0.001* | *p*=1.000 | *p*<0.001* |
| (20N,2.5cm) | $F=20.06$ $p<0.001$* | *p*<0.001* | *p*<0.001* | *p*<0.001* | *p*=1.000 | *p*=1.000 | *p*=1.000 |
| (20N,5.0cm) | $F=5.79$ $p<0.001$* | *p*<0.001* | *p*=1.000 | *p*=1.000 | *p*=0.102 | *p*=0.005* | *p*=1.000 |
| (20N,7.5cm) | $F=18.83$ $p<0.001$* | *p*<0.001* | *p*=0.608 | *p*<0.001* | *p*<0.001* | *p*=0.414 | *p*=0.016* |
| (25N,2.5cm) | $F=10.62$ $p<0.001$* | *p*=0.015* | *p*<0.001* | *p*=0.042* | *p*=0.015* | *p*=1.000 | *p*=0.135 |
| (25N,5.0cm) | $F=12.22$ $p<0.001$* | *p*<0.001* | *p*=0.103 | *p*=0.854 | *p*<0.001* | *p*<0.001* | *p*=1.000 |
| (25N,7.5cm) | $F=31.67$ $p<0.001$* | *p*<0.001* | *p*=0.418 | *p*<0.001* | *p*<0.001* | *p*=1.000 | *p*<0.001* |

*Damping*

*Effect of Target Force and Target Displacement: +X*

Supplementary Table 4 - Pairwise comparisons of damping estimates in +X direction with respect to target force and target displacement. The table reports the Bonferroni adjusted p-values for multiple comparisons. The significance was set to p=0.05. The left part of the table reports the comparisons between target displacements at the different force levels. The right part of the table reports the comparison between target forces at different displacement levels. The asterisk * denotes a significant difference.

|  | d=2.5cm/5.0cm | d=5.0/7.5cm | d=2.5cm/7.5cm |  | F=15N/20N | F=20N/25N | F=15N/25N |
| --- | --- | --- | --- | --- | --- | --- | --- |
| F=15N | *p*<0.001* | *p*<0.001* | *p*=0.842 | **d=2.5cm** | *p*=1.000 | *p*=0.021* | *p*=0.110 |
| F=20N | *p*=0.011* | *p*=0.005* | *p*<0.001* | **d=5.0cm** | *p*<0.001* | *p*<0.001* | *p*=1.000 |
| F=25N | *p*<0.001* | *p*=0.052 | *p*<0.001* | **d=7.5cm** | *p*<0.001* | *p*<0.001* | *p*<0.001* |

*Effect of Target Force and Target Displacement: -X*

Supplementary Table 5 - Pairwise comparisons of damping estimates in -X direction with respect to target force and target displacement. The table reports the Bonferroni adjusted p-values for multiple comparisons. The significance was set to p=0.05. The left part of the table reports the comparisons between target displacements at the different force levels. The right part of the table reports the comparison between target forces at different displacement levels. The asterisk * denotes a significant difference.

|  | d=2.5cm/5.0cm | d=5.0/7.5cm | d=2.5cm/7.5cm |  | F=15N/20N | F=20N/25N | F=15N/25N |
| --- | --- | --- | --- | --- | --- | --- | --- |
| F=15N | *p*<0.001* | *p*<0.001* | *p*=1.000 | **d=2.5cm** | *p*=0.347 | *p*=1.000 | *p*=0.377 |
| F=20N | *p*<0.001* | *p*<0.001* | *p*<0.001* | **d=5.0cm** | *p*=0.005* | *p*<0.001* | *p*<0.001* |
| F=25N | *p*<0.001* | *p*=0.020* | *p*<0.001* | **d=7.5cm** | *p*<0.001* | *p*<0.001* | *p*=0.001* |

*Effect of Target Force and Target Displacement: +Y*

Supplementary Table 6 - Pairwise comparisons of damping estimates in +Y direction with respect to target force and target displacement. The table reports the Bonferroni adjusted p-values for multiple comparisons. The significance was set to p=0.05. The left part of the table reports the comparisons between target displacements at the different force levels. The right part of the table reports the comparison between target forces at different displacement levels. The asterisk * denotes a significant difference.

|  | d=2.5cm/5.0cm | d=5.0/7.5cm | d=2.5cm/7.5cm |  | F=15N/20N | F=20N/25N | F=15N/25N |
| --- | --- | --- | --- | --- | --- | --- | --- |
| F=15N | *p*<0.001* | *p=*0.188 | *p*<0.001* | **d=2.5cm** | *p*=0.020* | *p*=0.554 | *p*<0.001* |
| F=20N | *p*<0.001* | *p*<0.001* | *p*<0.001* | **d=5.0cm** | *p*<0.001* | *p*=0.581 | *p*<0.001* |
| F=25N | *p*<0.001* | *p*<0.001* | *p*<0.001* | **d=7.5cm** | *p*<0.001* | *p*=0.087 | *p*<0.001* |

*Effect of Target Force and Target Displacement: -Y*

Supplementary Table 7 - Pairwise comparisons of damping estimates in -Y direction with respect to target force and target displacement. The table reports the Bonferroni adjusted p-values for multiple comparisons. The significance was set to p=0.05. The left part of the table reports the comparisons between target displacements at the different force levels. The right part of the table reports the comparison between target forces at different displacement levels. The asterisk * denotes a significant difference.

|  | d=2.5cm/5.0cm | d=5.0/7.5cm | d=2.5cm/7.5cm |  | F=15N/20N | F=20N/25N | F=15N/25N |
| --- | --- | --- | --- | --- | --- | --- | --- |
| F=15N | *p*=0.714 | *p*=0.157 | *p*=1.000 | **d=2.5cm** | *p*=0.594 | *p*=0.025* | *p*=1.000 |
| F=20N | *p*<0.001* | *p*<0.001* | *p*<0.001* | **d=5.0cm** | *p*=0.042* | *p*<0.001* | *p*<0.001* |
| F=25N | *p*<0.001* | *p*=0.016* | *p*<0.001* | **d=7.5cm** | *p*<0.001* | *p*<0.001* | *p*<0.001* |

*Effect of Direction*

Supplementary Table 8 – The table reports the repeated measures ANOVA results (second column) and the post-hoc pairwise comparisons of damping estimates with respect to direction. The pair-wise comparisons were Bonferroni adjusted for multiple comparisons. The significance was set to p=0.05. The asterisk * denotes a significant difference.

|  | ANOVA | (+X, -X) | (+X, +Y) | (+X, -Y) | (-X, +Y) | (-X, -Y) | (+Y, -Y) |
| --- | --- | --- | --- | --- | --- | --- | --- |
| (15N,2.5cm) | $F=49.55$ $p<0.001$* | *p*=1.000 | *p*<0.001* | *p*=0.004* | *p*<0.001* | *p*=0.007* | *p*<0.001* |
| (15N,5.0cm) | $F=93.59$ $p<0.001$* | *p*<0.001* | *p*<0.001* | *p*<0.001* | *p*<0.001* | *p*<0.001* | *p*=0.002* |
| (15N,7.5cm) | $F=12.56$ $p<0.001$* | *p*=1.000 | *p*<0.001* | *p*<0.001* | *p*=0.001* | *p*<0.001* | *p*=1.000 |
| (20N,2.5cm) | $F=31.56$ $p<0.001$* | *p*=1.000 | *p*<0.001* | *p*<0.001* | *p*<0.001* | *p*<0.001* | *p*<0.001* |
| (20N,5.0cm) | $F=102.67$ $p<0.001$* | *p*=0.304 | *p*<0.001* | *p*<0.001* | *p*<0.001* | *p*<0.001* | *p*<0.001* |
| (20N,7.5cm) | $F=53.05$ $p<0.001$* | *p*=0.003* | *p*<0.001* | *p*<0.001* | *p*<0.001* | *p*<0.001* | *p*=0.025* |
| (25N,2.5cm) | $F=31.35$ $p<0.001$* | *p*=0.006* | *p*<0.001* | *p*=1.000 | *p*<0.001* | *p*<0.001* | *p*<0.001* |
| (25N,5.0cm) | $F=51.22$ $p<0.001$* | *p*=1.000 | *p*<0.001* | *p*<0.001* | *p*<0.001* | *p*<0.001* | *p*<0.001* |
| (25N,7.5cm) | $F=67.42$ $p<0.001$* | *p*=1.000 | *p*<0.001* | *p*<0.001* | *p*<0.001* | *p*<0.001* | *p*<0.001* |

*Mass*

*Effect of Target Force and Target Displacement: +X*

Supplementary Table 9 - Pairwise comparisons of mass estimates in +X direction with respect to target force and target displacement. The table reports the Bonferroni adjusted p-values for multiple comparisons. The significance was set to p=0.05. The left part of the table reports the comparisons between target displacements at the different force levels. The right part of the table reports the comparison between target forces at different displacement levels. The asterisk * denotes a significant difference.

|  | d=2.5cm/5.0cm | d=5.0/7.5cm | d=2.5cm/7.5cm |  | F=15N/20N | F=20N/25N | F=15N/25N |
| --- | --- | --- | --- | --- | --- | --- | --- |
| F=15N | *p*<0.001* | *p*<0.001* | *p*=1.000 | **d=2.5cm** | *p*=0.595 | *p*=0.003* | *p*<0.001* |
| F=20N | *p*<0.001* | *p*=0.657 | *p*<0.001* | **d=5.0cm** | *p*=0.153 | *p*=0.277 | *p*=0.004* |
| F=25N | *p*<0.001* | *p*=1.000 | *p*<0.001* | **d=7.5cm** | *p*<0.001* | *p*=0.203 | *p*<0.001* |

*Effect of Target Force and Target Displacement: -X*

Supplementary Table 10 - Pairwise comparisons of mass estimates in -X direction with respect to target force and target displacement. The table reports the Bonferroni adjusted p-values for multiple comparisons. The significance was set to p=0.05. The left part of the table reports the comparisons between target displacements at the different force levels. The right part of the table reports the comparison between target forces at different displacement levels. The asterisk * denotes a significant difference.

|  | d=2.5cm/5.0cm | d=5.0/7.5cm | d=2.5cm/7.5cm |  | F=15N/20N | F=20N/25N | F=15N/25N |
| --- | --- | --- | --- | --- | --- | --- | --- |
| F=15N | p<0.001* | p<0.001* | p=0.013* | **d=2.5cm** | p=0.036* | p<0.001* | p=0.232 |
| F=20N | p<0.001* | p=0.013* | p<0.001* | **d=5.0cm** | p=0.165 | p=1.000 | p=0.496 |
| F=25N | p<0.001* | p=0.019* | p<0.001* | **d=7.5cm** | p<0.001* | p<0.001* | p<0.001* |

*Effect of Target Force and Target Displacement: +Y*

Supplementary Table 11 - Pairwise comparisons of mass estimates in +Y direction with respect to target force and target displacement. The table reports the Bonferroni adjusted p-values for multiple comparisons. The significance was set to p=0.05. The left part of the table reports the comparisons between target displacements at the different force levels. The right part of the table reports the comparison between target forces at different displacement levels. The asterisk * denotes a significant difference.

|  | d=2.5cm/5.0cm | d=5.0/7.5cm | d=2.5cm/7.5cm |  | F=15N/20N | F=20N/25N | F=15N/25N |
| --- | --- | --- | --- | --- | --- | --- | --- |
| F=15N | *p*<0.001* | *p*=0.724 | *p*<0.001* | **d=2.5cm** | *p*=0.099 | *p*<0.001* | *p*<0.001* |
| F=20N | *p*<0.001* | *p*=0.711 | *p*<0.001* | **d=5.0cm** | *p*=1.000 | *p*=0.562 | *p*=0.420 |
| F=25N | *p*<0.001* | *p*=0.265 | *p*<0.001* | **d=7.5cm** | *p*=0.079 | *p*=0.378 | *p*<0.001* |

*Effect of Target Force and Target Displacement: -Y*

Supplementary Table 12 - Pairwise comparisons of mass estimates in -Y direction with respect to target force and target displacement. The table reports the Bonferroni adjusted p-values for multiple comparisons. The significance was set to p=0.05. The left part of the table reports the comparisons between target displacements at the different force levels. The right part of the table reports the comparison between target forces at different displacement levels. The asterisk * denotes a significant difference.

|  | d=2.5cm/5.0cm | d=5.0/7.5cm | d=2.5cm/7.5cm |  | F=15N/20N | F=20N/25N | F=15N/25N |
| --- | --- | --- | --- | --- | --- | --- | --- |
| F=15N | *p*<0.001* | *p*<0.001* | *p*=1.000 | **d=2.5cm** | *p*=1.000 | *p*=0.008* | *p*=0.027* |
| F=20N | *p*<0.001* | *p*=0.035* | *p*<0.001* | **d=5.0cm** | *p*=0.875 | *p*=1.000 | *p*=0.321 |
| F=25N | *p*<0.001* | *p*=0.007* | *p*<0.001* | **d=7.5cm** | *p*=0.064 | *p*<0.001* | *p*<0.001* |

*Effect of Direction*

Supplementary Table 13 - The table reports the repeated measure ANOVA results (second column) and the post-hoc pairwise comparisons of mass estimates with respect to direction. The pair-wise comparisons were Bonferroni adjusted for multiple comparisons. The significance was set to p=0.05. The asterisk * denotes a significant difference.

|  | ANOVA | (+X, -X) | (+X, +Y) | (+X, -Y) | (-X, +Y) | (-X, -Y) | (+Y, -Y) |
| --- | --- | --- | --- | --- | --- | --- | --- |
| (15N,2.5cm) | $F=245.49$ $p<0.001$* | *p*<0.001* | *p*<0.001* | *p*<0.001* | *p*<0.001* | *p*<0.001* | *p*<0.001* |
| (15N,5.0cm) | $F=234.64$ $p<0.001$* | *p*=0.017* | *p*<0.001* | *p*<0.001* | *p*<0.001* | *p*<0.001* | *p*<0.001* |
| (15N,7.5cm) | $F=174.88$ $p<0.001$* | *p*<0.001* | *p*<0.001* | *p*<0.001* | *p*<0.001* | *p*<0.001* | *p*<0.001* |
| (20N,2.5cm) | $F=261.62$ $p<0.001$* | *p*<0.001* | *p*<0.001* | *p*<0.001* | *p*<0.001* | *p*<0.001* | *p*=0.005* |
| (20N,5.0cm) | $F=263.37$ $p<0.001$* | *p*=0.001* | *p*<0.001* | *p*<0.001* | *p*<0.001* | *p*<0.001* | *p*<0.001* |
| (20N,7.5cm) | $F=209.95$ $p<0.001$* | *p*<0.001* | *p*<0.001* | *p*<0.001* | *p*<0.001* | *p*<0.001* | *p*<0.001* |
| (25N,2.5cm) | $F=228.07$ $p<0.001$* | *p*<0.001* | *p*<0.001* | *p*<0.001* | *p*<0.001* | *p*<0.001* | *p*=0.138 |
| (25N,5.0cm) | $F=279.34$ $p<0.001$* | *p*<0.001* | *p*<0.001* | *p*<0.001* | *p*<0.001* | *p*<0.001* | *p*<0.001* |
| (25N,7.5cm) | $F=209.30$ $p<0.001$* | *p*=0.049* | *p*<0.001* | *p*<0.001* | *p*<0.001* | *p*<0.001* | *p*<0.001* |
